# Does a single session of transcranial direct current stimulation enhance both physical and psychological performance in national- or international-level athletes? A systematic review

**DOI:** 10.3389/fphys.2024.1365530

**Published:** 2024-06-19

**Authors:** Ying Yu, Xinbi Zhang, Michael A. Nitsche, Carmelo M. Vicario, Fengxue Qi

**Affiliations:** ^1^ Key Laboratory of Sport Training of General Administration of Sport of China, Beijing Sport University, Beijing, China; ^2^ Sports, Exercise and Brain Sciences Laboratory, Beijing Sport University, Beijing, China; ^3^ Department of Psychology and Neurosciences, Leibniz Research Centre for Working Environment and Human Factors, Dortmund, Germany; ^4^ University Clinic of Psychiatry and Psychotherapy and University Clinic of Child and Adolescent Psychiatry and Psychotherapy, Protestant Hospital of Bethel Foundation, University Hospital OWL, Bielefeld University, Bielefeld, Germany; ^5^ Department of Cognitive Sciences, Psychology, Education and Cultural Studies, University of Messina, Messina, Italy

**Keywords:** high-level athletes, physical performance, psychological performance, noninvasive brain stimulation, efficacy

## Abstract

Some studies showed that a single session of transcranial direct current stimulation (tDCS) has the potential of modulating motor performance in healthy and athletes. To our knowledge, previously published systematic reviews have neither comprehensively investigated the effects of tDCS on athletic performance in both physical and psychological parameters nor investigated the effects of tDCS on high-level athletes. We examined all available research testing a single session of tDCS on strength, endurance, sport-specific performance, emotional states and cognitive performance for better application in competition and pre-competition trainings of national- or international-level athletes. A systematic search was conducted in PubMed, Web of Science, EBSCO, Embase, and Scopus up until to June 2023. Studies were eligible when participants had sports experience at a minimum of state and national level competitions, underwent a single session of tDCS without additional interventions, and received either sham tDCS or no interventions in the control groups. A total of 20 experimental studies (224 participants) were included from 18 articles. The results showed that a single tDCS session improved both physical and psychological parameters in 12 out of the 18 studies. Of these, six refer to the application of tDCS on the motor system (motor cortex, premotor cortex, cerebellum), five on dorsolateral prefrontal cortex and two on temporal cortex. The most sensitive to tDCS are strength, endurance, and emotional states, improved in 67%, 75%, and 75% of studies, respectively. Less than half of the studies showed improvement in sport-specific tasks (40%) and cognitive performance (33%). We suggest that tDCS is an effective tool that can be applied to competition and pre-competition training to improve athletic performance in national- or international-level athletes. Further research would explore various parameters (type of sports, brain regions, stimulation protocol, athlete level, and test tasks) and neural mechanistic studies in improving efficacy of tDCS interventions.

**Systematic Review Registration:** https://www.crd.york.ac.uk/prospero/display_record.php?ID=CRD42022326989, identifier CRD42022326989.

## 1 Introduction

Athletes undergo systematic training over a long time to improve their athletic performance and achieve superior athletic results in competition. Athletic performance is multifactorial, including both physical and psychological aspects. It requires the development of not only muscular endurance, explosive power, motor coordination and quick and accurate strategic thinking (Colzato et al., 2016), but also stable emotional states and cognitive performance (Hagan et al., 2017). Athletes are often willing to apply top-tier training techniques to enhance athletic performance. Numerous studies have revealed that the brain is crucial for improving athletic performance beyond muscles, heart and lung (Noakes, 2011).

Transcranial direct current stimulation (tDCS) is a non-invasive brain stimulation technique that applies a constant weak direct electrical current using two or more electrodes placed on the scalp to facilitate or inhibit neural activity and/or excitability (Nitsche et al., 2003b). Classically, the effects of tDCS are polarity-dependent in that anodal tDCS (a-tDCS) enhances cortical excitability by subthreshold depolarization of the neural resting membrane potential, and cathodal tDCS (c-tDCS) reduces cortical excitability through hyperpolarization of the neural resting membrane potential (Nitsche and Paulus, 2000; Liebetanz et al., 2002; Stagg and Nitsche, 2011). The after-effects of tDCS last for up to 120 min, or longer, and have been linked to long-term potentiation or long-term depression (Nitsche and Paulus, 2001; Polania et al., 2012; Jamil et al., 2017). Besides, some studies showed that tDCS is an effective method for inducing sustained and widespread changes in regional neural activity and functional connectivity (Lang et al., 2005; Polania et al., 2012). tDCS is widely used in basic research, neuropsychiatric and neurological disorders to modulate cortical excitability, reorganize neuroplasticity, and improve motor performance (Fregni et al., 2006; Angius et al., 2017; Gold and Ciorciari, 2021; Yang et al., 2021; Hou et al., 2022). Therefore, it has attracted the attention of sports scientists. There have been substantial studies showing that tDCS applied over the primary motor cortex (M1), temporal cortex (TC), cerebellum (CB), premotor cortex (PMC) and dorsolateral prefrontal cortex (DLPFC) enhances crucial aspects of motor performance, including muscular strength (Maezawa et al., 2020; Vieira et al., 2020), endurance (Lattari et al., 2016; Ciccone et al., 2019), cognitive control (Wiegand et al., 2019), dynamic balance (Kaminski et al., 2016; Yosephi et al., 2018), and reaction speed (Denis et al., 2019) in healthy people and lower-level athletes.

The effects of tDCS on strength and endurance are more researched, but the results are mixed. The review of Holgado et al. (2019) reported a-tDCS had a small but positive effect on exercise performance, but the effects were not significantly moderated by type of outcome, electrode placement, muscles involved, number of sessions, or intensity and duration of the stimulation. A previous systematic review and meta-analysis reported a-tDCS leads to small and moderate effects on strength (maximal voluntary contraction) and endurance (time to task failure) in healthy young and old adults (Alix-Fages et al., 2019). The review of Machado et al. (2019) reported that a-tDCS but not c-tDCS over M1 improved exercise performance in cycling only in healthy adults. The review of Shyamali Kaushalya et al. (2021) reported that a-tDCS enhance running and cycling time in time to exhaustion tasks but no improved in endurance time trial or sprint tasks in healthy people. The review of Chinzara et al. (2022) reported that tDCS has the potential to be used as an ergogenic aid in physical endurance, muscular strength, and visuomotor skills in healthy adults. This controversy in reviews might be attributed to different brain region targets, stimulation protocols, test tasks, and motor abilities in participants.

Some studies showed that tDCS effectively improves sport-specific performance, such as golf putting (Zhu et al., 2015) and basketball performance (shooting precision, specific dribbling, and agility) in healthy people (Veldema et al., 2021). A recent systematic review and meta-analysis reported that a single anodal tDCS session can enhance sport-specific motor performance in healthy adult athletes who have been regularly participating in organized sports for at least 2 years (Maudrich et al., 2022). However, some studies yielded inconsistent results (Valenzuela et al., 2019; Penna et al., 2021; Moreira et al., 2022). Achieving high-level of athletic performance is often believed to demand a minimum of a decade or 10,000 h of practice, based on the time dedicated to practicing a task (Ericsson KA and Tesch-Römer, 1993). According to McKay et al. (McKay et al., 2022) ‘s classification of sports levels, athletes above tier 3 (Highly Trained/National Level, Elite/International Level and World Class) in studies could provide reliable performance outcomes and suited to both laboratory- and field-based research. The brains of high-level athletes have different characteristics compared with the general population and lower-level athletes (Nakata et al., 2010), such as higher neural excitability (Wright et al., 2010; Callan and Naito, 2014; Monda et al., 2017) and specific neural responses during exercise (Del Percio et al., 2010; Casanova et al., 2013; Ermutlu et al., 2015). Whether a single session of tDCS can apply to competition and pre-competition training in national- or international-level athletes remains uncertain. More importantly, psychological parameters such as emotional state and cognitive performance are important for athletic performance under intense conditions (Hagan et al., 2017; Valenzuela et al., 2019; Fortes et al., 2022). Emotional state had proven a relationship with athletic performance and change of sporting success (Zandi and Rad, 2013; Brandt et al., 2017). Cognitive performance such as inhibitory control, visual search and decision-making are crucial for accuracy and consistency of movements, as well as efficiently processing the surrounding information to adopt the correct action in a given situation (Chiu et al., 2017). Yet previous review studies focused only on the effects of tDCS on motor and sport-specific performance in healthy people and athletes of all levels, with less attention paid to cognitive performance and emotional states. Whether a single session of tDCS improved athletic performance in high-level athletes and in what aspects it is effective is still inconclusive. Therefore, we carried out a systematic review to comprehensively analyze the effects of a single session of tDCS on athletic performance in strength, endurance, sport-specific performance, emotional states and cognitive performance in national- or international-level athletes, in order to improve the efficacy of tDCS on high-level athletes.

## 2 Methods

A systematic literature review was conducted according to the Preferred Reporting Items for Systematic Reviews and Meta-Analyses (PRISMA) guidelines (Liberati et al., 2009). Details of the review protocol were registered on PROSPERO on April 2022 (CRD42022326989). A meta-analysis was not conducted due to high measurement and methodological heterogeneity in the selected studies.

### 2.1 Literature search

The literature search was performed until June 2023 by two independent researchers (Y.Y., Z.X.B.) using the PubMed, Web of Science, EBSCO, Embase, and Scopus databases, and the keywords (“tDCS” OR “transcranial Direct Current Stimulation”) AND (“athlete” OR “player” OR “sport” OR “athletic performance” OR “psychological performance” OR “physical performance” OR “cognitive performance” OR “cognitive function”). Additional relevant studies were also scanned in the references of the retrieved literature to broaden the search.

### 2.2 Study inclusion and exclusion criteria

Studies were included according to the “participants, intervention, comparator, outcomes, study design” inclusion criteria: 1) healthy athletes in highly trained/national level and elite/international level (McKay et al., 2022); 2) intervention consisting of a single tDCS session; 3) sham tDCS or no interventions as control groups; 4) inclusion of all detailed test results; and 5) randomized controlled trials with crossover or parallel group designs.

The exclusion criteria were: 1) no specification of the level of athletes; 2) other interventions during the experiment; and 3) non-English language, review papers, conference abstracts, study protocols, and papers without relevant experimental data.

### 2.3 Study selection and risk-of-bias assessment

The literature screening was conducted independently by two researchers based on the inclusion and exclusion criteria. A third researcher was consulted in case of disagreement. The Cochrane Risk of Bias assessment tool assessed bias in the included studies (Habay et al., 2021). Quality assessment included random sequence generation, allocation concealment, blinding of participants and personnel, blinding of outcome assessment, incomplete outcome data bias, selective reporting, and other biases. Each component was rated as having a “high”, “low”, or “unclear” risk of bias. The Review Manager 5.4.1 software (Cochrane Collaboration, Oxford, UK) was used.

## 3 Results

### 3.1 Search results

**FIGURE 1:**
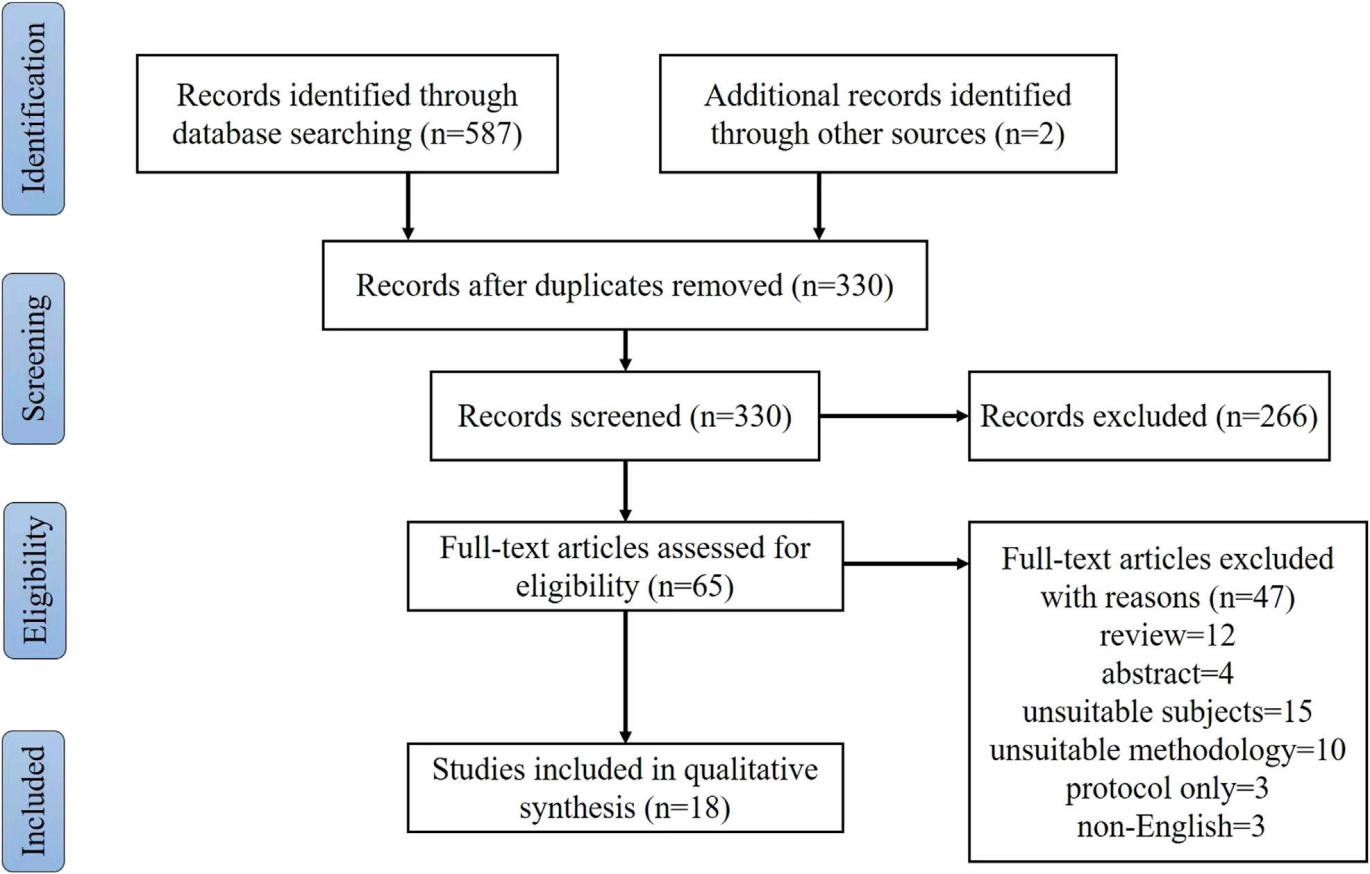
Selection of studies.

A total of 589 articles (PubMed, 208; Web of Science, 92; EBSCO, 87; Embase, 70; Scopus, 130; other sources, 2) were found in the systematic literature search. After the removal of duplicate articles, 330 articles were screened based on their title and abstract, of which 266 articles were excluded. After full-text assessment, 47 records were excluded. Finally, 18 articles were eligible for the systematic review. The literature search process is shown in Figure 1.

### 3.2 Quality and risk-of-bias assessment

**FIGURE 2:**
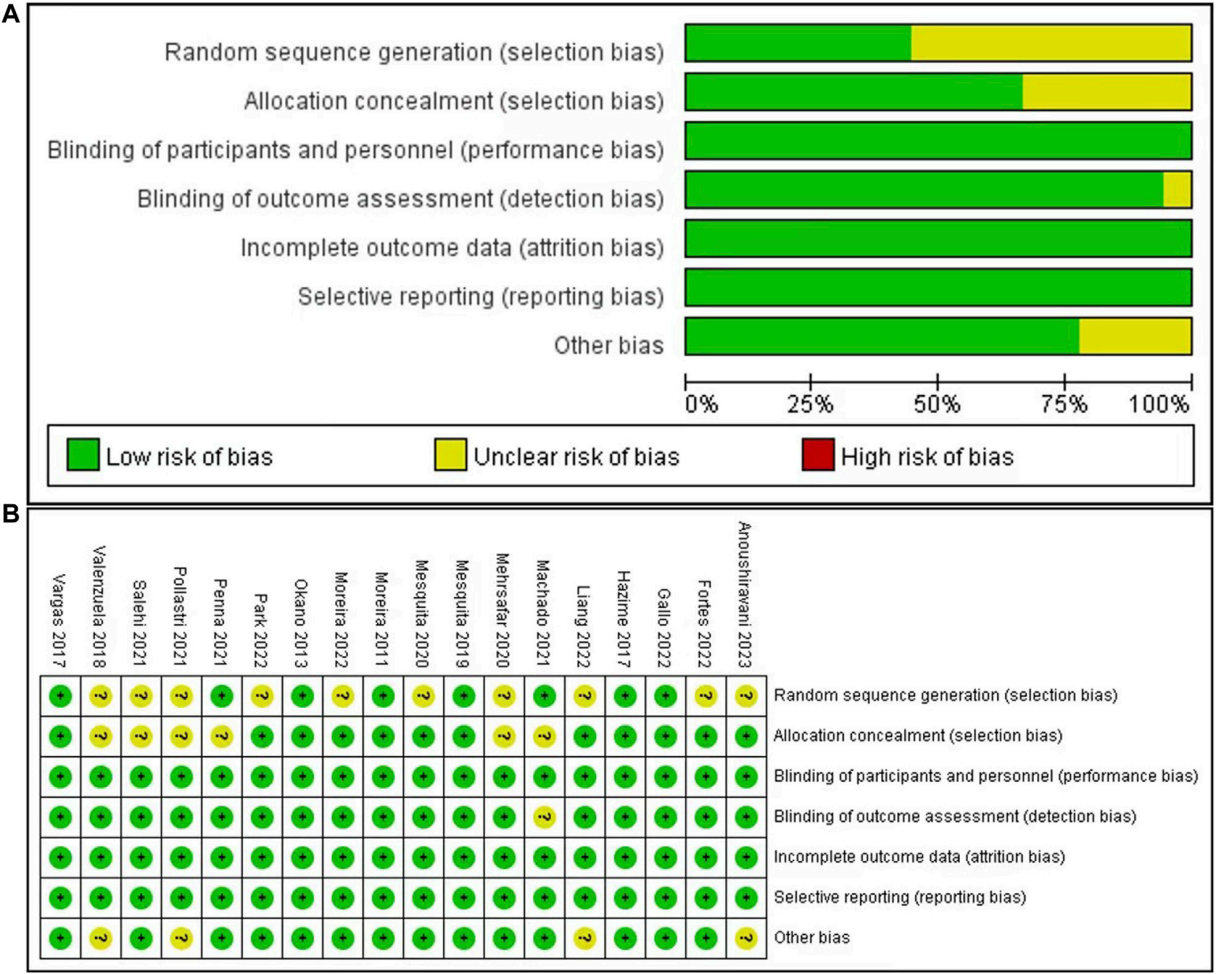
Quality and risk-of-bias assessment. Risk of bias graph **(A)**: review authors’ judgments about each risk of bias item presented as percentages across all included studies; and risk of bias summary **(B)**: review authors’ judgments about each risk of bias item for each included study.

The overall quality of the included studies was high, with all studies using a randomized controlled experimental design. However, 12 of the included studies showed an unclear risk of selection bias, due to unclear random sequence generation in 10 articles and unclear allocation concealment in six articles. One study showed an unclear risk with respect to blinding of outcome assessment. Four studies showed an unclear risk of other biases. The risk-of-bias graphs and a summary are presented in Figure 2.

### 3.3 Study characteristics and result synthesis

An overview of literature characteristics is given in Table 1. All studies used crossover designs with a sham condition as a comparator. In total, 224 athletes were included in 18 studies, with 156 men (69.64%) and 68 women (30.36%). The mean age of the participants ranged from 16.10 ± 0.90 years (Vargas et al., 2018) to 33.00 ± 9.00 years (Okano et al., 2015). The average age of the subjects in one study conducted by Liang et al. (2022) was unknown. All studies used sham tDCS as a control condition. The studies covered handball, soccer, shooting, swimming, basketball, gymnastic, volleyball, triathlon, rowing, taekwondo, archery, and cycling. All subjects had sports experience at least state- and national-level competitions. All experimental groups used a-tDCS, while two studies used both, anodal/cathodal stimulation (Mehrsafar et al., 2020; Salehi et al., 2022). With the exception of eight studies applying a cephalic montage of the return electrode, all examined studies applied an extracephalic montage of the return electrode. Regarding the tDCS application protocol, all studies opted for the offline modality (testing after stimulation), with five studies including also the online stimulation modality (testing during stimulation) (Hazime et al., 2017; Vargas et al., 2018; Fortes et al., 2022; Moreira et al., 2022; Anoushiravani et al., 2023). Three studies out of 18 studies did not measure side effects of the tDCS intervention. None of the studies reported any adverse effects or injuries after the intervention and the common sensations were itching and tingling. The basic characteristics of the literature are shown in Tables 1, 2.

**TABLE 1:** Characteristics of the studies.

References	Sports	Gender	Age	Sports experience	Timing	Model	Design
Okano et al. (2015)	Cycling	10M	33.00 ± 9.00	Nation level	Offline	a-tDCS	Crossover
Hazime et al. (2017)	Handball	8F	19.65 ± 2.55	State and national competitions	Online/Offline	a-tDCS	Crossover
Mesquita et al. (2019a)	Taekwondo	12M7F	19.00 ± 3.00	Nation level	Offline	a-tDCS	Crossover
Vargas et al. (2018)	Soccer	20F	16.10 ± 0.90	State and national competitions	Online/Offline	a-tDCS	Crossover
Valenzuela et al. (2019)	Triathlon	8M	22.00 ± 2.00	International competitions	Offline	a-tDCS	Crossover
Mesquita et al. (2020)	Taekwondo	8M4F	19.00	National or international tournaments	Offline	a-tDCS	Crossover
Mehrsafar et al. (2020)	Archery	12M	26.51 ± 2.31	National level	Offline	a/c-tDCS	Crossover
Moreira et al. (2021)	Soccer	12M	19.00 ± 1.00	Professional Football Club Players	Offline	a-tDCS	Crossover
Pollastri et al. (2021)	Cycling	8M	20.00 ± 1.50	International competitions	Offline	a-tDCS	Crossover
Machado et al. (2021)	Cycling, Rowing	12M	29.40 ± 7.30	State, national or world-class competitions	Offline	a-tDCS	Crossover
Salehi et al. (2022)	Swimming	15M	23.00 ± 1.00	State and national competitions	Offline	a/c-tDCS	Crossover
Penna et al. (2021)	Swimming	10M	30.00 ± 6.00	National level	Offline	a-tDCS	Crossover
Liang et al. (2022)	Rowing	8F	NR	National level	Offline	a-tDCS	Crossover
Park et al. (2022)	Volleyball	13F	21.92 ± 2.81	Professional Volleyball Club Players	Offline	a-tDCS	Crossover
Moreira et al. (2022)	Basketball	8F	25.00 ± 8.00	Professional Basketball Players	Online/Offline	a-tDCS	Crossover
Gallo et al. (2022)	Cycling	12M	21.3 ± 4.30	Nation level	Offline	a-tDCS	Crossover
Fortes et al. (2022a)	Basketball	20M	24.77 ± 4.2	National level	Online/Offline	a-tDCS	Crossover
Anoushiravani et al. (2023)	Gymnastic	17M	21.05 ± 2.04	National competitions	Online/Offline	a-tDCS	Crossover

Abbreviations: M = male; F = female; NR, not reported; a-tDCS, anodal tDCS; c-tDCS, cathodal tDCS.

**TABLE 2:** Overview of studies investigating a single session of transcranial direct current stimulation for improving athletic performance in elite athletes.

References		Stimulus(S) Reference(R)	Electrode size [cm^2^]	Intensity [mA]	Duration [mins]	Indicators	Main outcomes
Strength							
Hazime et al.		S:M1 (C3 or C4) R:IOC	S = R = 35	2.0	20	MVIC	a-tDCS improved MVIC of shoulder rotator
Vargas et al.		S:M1 (C3 or C4) R:COC	S = R = 35	2.0	20	MVIC	a-tDCS improved MVIC of external and internal knee extensor
Mesquita et al.		S:Bilateral M1 (C3, C4) R:IS	S = 35 R = 25	1.5	15	CMJ	No significant difference in CMJ
Park et al.		S:M1 (Cz) R:M1 (C5, C6)	S = R = 28	2.0	20	1RM, CMJ	No significant difference in CMJ, bench-press and back-squat 1 RM
Anoushiravani et al.		S: Bilateral PMC R: COC	S = R = 35	2.0	20	BJT, MVIC	a-tDCS improved BJT, MVIC of all upper body muscles
		S: Bilateral CB R: COC	S = R = 35	2.0	20		a-tDCS improved MVIC of right deltoid and trapezius, left biceps and pectoralis muscle
Endurance							
Okano et al.		S:Left TC (T3) R:COC	S = R = 35	2.0	20	MIT	a-tDCS improved PPO
Pollastri et al.		S:Bilateral DLPFC (F3, F4) R:Fp1, F7, C3, Fp2, F8, C4	S = R = 3.14	1.5	20	15 km TT	a-tDCS improved PPO and total time
Machado et al.		S:M1 (Cz, C1, C2) R:C3, C4, FC1, FC2, P1, P2	NR	2.4	20	TTE at 80% PPO	No significant difference
		S:M1 (Cz) R: Occipital protuberance	S = 36 R = 35	2.0	20		
Gallo et al.		S:Bilateral DLPFC (F3, F4) R:Fp1, F7, C3, Fp2, F8, C4	S = R = 3.14	1.5	20	2 km TT	a-tDCS improved total time
Sport-specific performance							
Salehi et al.	S:Left DLPFC (F3) R:COC (Fp2)		S = R = 35	2.0	20	50-m swimming test	a-tDCS improved total time
Valenzuela et al.	S:M1 (C3) R:COC (Fp2)		S = R = 25	2.0	20	800-m swimming test	No significant difference
Penna et al.	S:Left TC (T3) R:IS		S = R = 35	2.0	30	800-m swimming test	No significant difference
Moreira et al.	S:Left DLPFC (F3) R:Right DLPFC (F4)		S = R = 35	2.0	20	Shooting task	No significant difference
Liang et al.	S:M1 (Cz) R:C5, C6		S = R = 24	2.2	20	5 km rowing	No significant difference in speed and power
Mesquita et al.	S:Bilateral M1 (C3, C4) R:IS		S = 35 R = 25	1.5	15	FSKT	a-tDCS worsens the total number of kicks
Park et al.	S:M1 (Cz) R:C5, C6		S = R = 28	2.0	20	Spike task	a-tDCS improved spike speed
Mesquita et al.	S:Bilateral M1 (C3, C4) R:IS		S = 35 R = 25	1.5	15	PSTT	No significant difference in kicking frequency
Anoushiravani et al.	S: Bilateral PMC R: COC		S = R = 35	2.0	20	BHST, SLHT,DLCT, ASFT,SRT,DPBT	a-tDCS improved SLHT, BHST, DPBT
	S: Bilateral CB R: COC		S = R = 35	2.0	20		a-tDCS improved SLHT
Emotional state							
Valenzuela et al.	S:M1 (C3) R:COC (Fp2)		S = R = 25	2.0	20	BMS	a-tDCS improved vigor
Mehrsafar et al.	S:Left DLPFC (F3) R:Right DLPFC (F4)		S = R = 25	2.0	20	BMS, CSAI-2	a-tDCS improved vigor, decreased tension, fatigue and anxiety
Moreira et al.	S:Left DLPFC (F3) R:Right DLPFC (F4)		S = R = 35	2.0	20	WBQ	a-tDCS improved total score
Machado et al.	S:M1 (Cz, C1, C2) R:C3, C4, FC1, FC2, P1, P2		NR	2.4	20	BMS	No significant difference in total score
	S:M1 (Cz) R: Occipital protuberance		S = 36 R = 35	2.0	20		
Cognitive performance							
Moreira et al.	S:Left DLPFC (F3) R:Right DLPFC (F4)		S = R = 35	2.0	20	Stroop task	No significant difference in RT and accuracy
Gallo et al.	S:Bilateral DLPFC (F3, F4) R:Fp1, F7, C3, Fp2, F8, C4		S = R = 3.14	1.5	20	Stroop task	No significant difference in RT and accuracy
Fortes et al.	S:Middle TC (CP5) R:Visual cortex (Oz)		S = R = 25	2.0	30	Visuomotor task Decision-making	a-tDCS improved RT and accuracy in decision-making, and RT in visuomotor task

The effects of tDCS on improving athletic performance were categorized into strength, endurance, sport-specific performance, emotional states, and cognitive performance. Abbreviations: a-tDCS, anodal tDCS; FSKT, frequency speed of kick test; PSTT, progressive specific taekwondo test; MVIC, maximal voluntary isometric contraction; CMJ, countermovement jump; TTE, time to exhausted test; MIT, maximal incremental test; TT, time trail; PPO, peak power output; 1RM, 1 repetition maximum; BJT, broad jump test; SLHT, straddle lift to handstand test; DLCT, double legs circle test; BHST, back hang scale test; ASFT, active shoulder flexibility test; SRT, sit and reach test; DPBT, dips on parallel bars test; WBQ, Wellbeing Questionnaire; BMS, brunel mood scale; CSAI-2, competitive state anxiety inventory second edition; RT, reaction time; cm^2^ = square centimeter; mA = milliamps; mins, minutes; M1 = primary motor cortex; DLPFC, dorsolateral prefrontal cortex; TC, temporal cortex; PMC, premotor cortex; CB, cerebellum; COC, contralateral orbitofrontal cortex; IOC, ipsilateral orbitofrontal cortex; IS, ipsilateral shoulder; S = stimulating electrode; R = reference electrode; NR, not reported; F = female; M = male.

**FIGURE 3:**
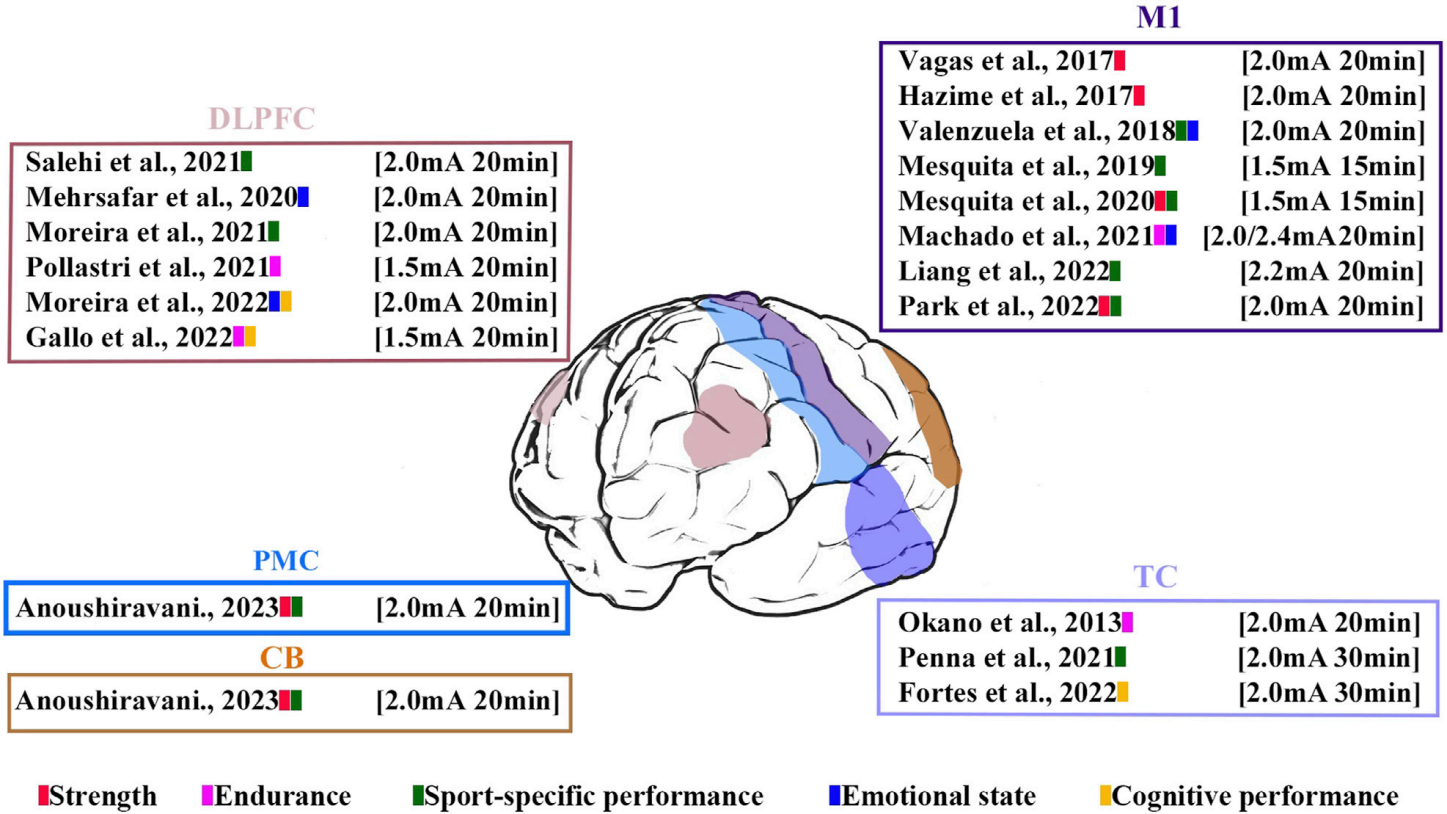
Visualization of stimulated time, site, current intensity and performance in the analyzed studies. The color bars represent athletic performance involved in analyzed studies.

These studies contained five stimulation regions: M1 (44.44%), DLPFC (33.33%), PMC (5.5%), CB (5.5%), and TC (16.67%). Five studies used bilateral anodal stimulation protocols (Mesquita et al., 2019b; Mesquita et al., 2020; Pollastri et al., 2021; Gallo et al., 2022; Anoushiravani et al., 2023), and the remaining studies used unilateral stimulation. Three studies used high-definition tDCS (HD-tDCS), and the other studies used a conventional tDCS setup using two electrodes (anode and cathode). Regarding the HD-tDCS setups, two used two sets of 3 × 1 electrodes (i.e., one anode and three cathodes) with 3.14-cm2 circular electrodes (Pollastri et al., 2021; Gallo et al., 2022), and Machado et al. (Machado et al., 2021) adopted a 6 × 3 (i.e., three anodes and six cathodes) ring configuration. Studies used stimulation durations of 15–30 min. The current intensity was 1.5 mA (Pollastri et al., 2021), 2.0 mA (Okano et al., 2015; Hazime et al., 2017; Vargas et al., 2018; Valenzuela et al., 2019; Mesquita et al., 2020; Machado et al., 2021; Penna et al., 2021; Moreira et al., 2022; Park et al., 2022; Salehi et al., 2022; Anoushiravani et al., 2023), 2.2 mA (Liang et al., 2022), and 2.4 mA (Machado et al., 2021). The electrode size varied between 3.14 cm2 and 36 cm2. The electrode size for HD-tDCS in the study by Machado et al. (2021) was not reported. The stimulation times, sites, and current intensities are shown in Figure 3.

The effects of tDCS on improving athletic performance were categorized into the following domains: strength (5 studies), endurance (4 studies), sport-specific performance (9 studies), emotional states (4 studies), and cognitive performance (3 studies).

Regarding muscle strength measurements, five studies used maximal voluntary isometric contraction (MVIC), the countermovement jump test (CMJ), broad jump test (BJT), and one-repetition maximum loads to measure the effects of tDCS in soccer, handball, taekwondo, gymnastic, and volleyball athletes (Hazime et al., 2017; Vargas et al., 2018; Mesquita et al., 2019a; Park et al., 2022; Anoushiravani et al., 2023). Among them, three studies (60%) found improvements. Vargas et al. (2018) and Hazime et al. (2017) found that 2-mA a-tDCS (0.0571 mA/cm2) over M1 (electrodes C3 or C4; International 10–20 System) for 20 min improved the MVIC of knee extensors in soccer players and external and internal shoulder rotators in handball players during stimulation, these effects lasted for 60 min. Anoushiravani et al. (2023) reported that 2-mA a-tDCS (0.0571 mA/cm2) improved BJT performance and MVIC of all tested upper body muscles when applied over the PMC for 20 min, and MVIC of the right deltoid and trapezius, and left biceps and pectoralis muscles when applied over the CB for 20 min. Park et al. (2022) reported no significant improvements of CMJ performance, bench press performance, and back squat one-repetition maximum loads in volleyball players by 2-mA a-tDCS (0.0714 mA/cm2) over M1 (Cz) for 20 min. Mesquita et al. (2019a) found that 1.5-mA a-tDCS (0.0428 mA/cm2) for 15 min applied over M1 bilaterally (C3 and C4) did not have significant improving effects on CMJ performance in taekwondo athletes.

Regarding endurance performance, four studies used peak power output (PPO) and the time in time-to-exhaustion test (TTE), time trial test (TT), or maximal incremental test (MIT) to assess the effects of tDCS in cyclists and rowers (Okano et al., 2015; Machado et al., 2021; Pollastri et al., 2021; Gallo et al., 2022). Among these studies, three (75%) reported positive influences. Okano et al. (Okano et al., 2015) showed that 2-mA a-tDCS (0.0571 mA/cm2) applied over the left TC (T3) for 20 min increased PPO in the MIT in cyclists. Gallo et al. and Pollastri et al. applied 1.5-mA HD-tDCS (0.238 mA/cm2) over the bilateral DLPFC (F3 and F4) for 20 min, and this stimulation protocol improved total time in 2-km TT, PPO and total time in 15-km PPO in cyclists compared with sham stimulation (Pollastri et al., 2021; Gallo et al., 2022). Machado et al. (2021) however report that neither 2.4-mA HD-tDCS over the M1 (Cz, C1, and C2) for 20 min nor 2-mA conventional a-tDCS over M1 (Cz) for 20 min affected total time in TTE at 80% PPO in cyclists and rowers compared with sham stimulation. The authors did not report the electrode sizes used for HD-tDCS, and the stimulus and reference electrode current densities were 0.055 mA/cm2 and 0.0571 mA/cm2, respectively, for conventional tDCS.

Regarding sport-specific performance, the test parameters in the nine studies included swimming speed (50 m and 800 m), 5 km rowing, basketball shooting accuracy, volleyball spike performance, gymnastic performance, the Progressive Specific Taekwondo Test, and the Taekwondo frequency speed of kick test (Mesquita et al., 2019b; Valenzuela et al., 2019; Mesquita et al., 2020; Penna et al., 2021; Liang et al., 2022; Moreira et al., 2022; Park et al., 2022; Salehi et al., 2022). Among them, only three studies reported positive effects of tDCS. Salehi et al. (2022) reported that 2-mA a-tDCS (0.0571 mA/cm2) applied over the left DLPFC (F3) for 20 min improved 50-m swimming speed in professional swimmers. Moreira et al. (2022) used identical tDCS parameters and found however no effects on shooting accuracy in basketball players. Moreover, neither Valenzuela et al.nor Penna et al. found improvements in the 800-m swimming test using 2-mA a-tDCS over M1 (C3) for 20 min and left TC (T3) for 30 min (Valenzuela et al., 2019; Penna et al., 2021). Park et al. (2022) showed that 2-mA a-tDCS (0.0714 mA/cm2) over M1 (Cz) for 20 min improved volleyball spike speed and consistency compared with sham stimulation. Mesquita et al. (2019b; 2020) reported that 1.5-mA a-tDCS over the bilateral M1 (C3 and C4) for 15 min did not improve performance of the Progressive Specific Taekwondo Test and worsened performance on the frequency speed of kick test in taekwondo athletes. Similarly, Liang et al. (2022) showed that 2.2-mA a-tDCS over M1 (Cz) for 20 min did not increase 5-km rowing speed. Anoushiravani et al. (2023) reported that 2-mA a-tDCS over the PMC for 20 min improved performance on the straddle lift to handstand test, back hang scale test, and dips on parallel bars test and that 2-mA a-tDCS over the CB for 20 min improved straddle lift to handstand test performance in gymnastic athletes.

Four studies investigated the effects of tDCS on emotional states, with testing parameters including the Brunel Mood Scale, Competitive State Anxiety Inventory Second Edition, and Well-Being Questionnaire in archers, soccer players, triathletes, cyclists, and rowers (Valenzuela et al., 2019; Mehrsafar et al., 2020; Machado et al., 2021; Moreira et al., 2021). Among these studies, three reported positive results. Mehrsafar et al. found that 2-mA (0.08 mA/cm2) a-tDCS over the left DLPFC (F3) for 20 min improved vigor and reduced tension and anxiety in archers (Mehrsafar et al., 2020). Moreira et al. (2021) used the same tDCS parameters and found that a-tDCS (0.0571 mA/cm2) improved wellbeing in soccer players after a match. Still, there were inconsistent results regarding the effects of tDCS applied over the M1 on emotional states. Valenzuela et al. (2019) showed that 2-mA a-tDCS over the M1 (C3) for 20 min improved the vigor of triathletes compared with sham stimulation. Machado et al. (2021) found that neither HD-tDCS nor conventional a-tDCS over M1 improved Brunel Mood Scale scores in cyclists and rowers.

Three studies investigated the effects of tDCS on cognitive performance using reaction time (RT) and accuracy in the Stroop task (Okano et al., 2015; Penna et al., 2021), visuomotor skill and decision-making task (Fortes et al., 2022) in basketball players and cyclists. Among them, one study reported positive results. Fortes et al. (2022) found that 2 mA a-tDCS (0.08 mA/cm2) over the middle TC (CP5) for 30 min improved RT and accuracy in visuomotor skills as well as RT in decision-making in basketball players. Moreira et al. (2022) did not find improvements in Stroop task RT and accuracy in basketball players using 2-mA a-tDCS over the left DLPFC (F3) for 20 min. Moreover, Gallo et al. (2022) showed that 1.5-mA HD-tDCS over the bilateral DLPFC (F3 and F4) for 20 min did not improve Stroop task RT and accuracy in cyclists.

## 4 Discussion

In the present study, we performed a systematic review of the literature on the effects of a single session of tDCS in national- or international-level athletes. Numerous studies and reviews have investigated the effects of tDCS on exercise performance such as strength and endurance in individuals with motor impairments and in healthy individuals (Alix-Fages et al., 2019; Holgado et al., 2019; Morya et al., 2019; Patel et al., 2019; Chinzara et al., 2022; Marinus et al., 2022), but the number of studies on athletes, particularly national- or international-level athletes, is limited. A recent systematic review and meta-analysis investigated a single anodal tDCS session on sport-specific motor performance in athletes participating regularly for at least 2 years (Maudrich et al., 2022). As far as we know, no reviews study has comprehensively investigated the effects of tDCS on athletic performance in both physical and psychological parameters in high-level athletes. Our studies included high-level athletes at tiers 3 (highly trained/national level) and four (international level), based on a recently proposed participant classification framework (McKay et al., 2022). Therefore, we examined 18 studies, involving 224 subjects in 12 sports disciplines. Our analysis showed that a single session of 1.5–2.4-mA tDCS with a duration of 15–30 min resulted in heterogeneous outcomes. The most sensitive to tDCS are strength (improvement in 67% of studies), endurance (improvement in 75% of studies), and emotional states (improvement in 75% of studies). It is interesting to note that the positive effects of tDCS on performance are significantly reduced in the case of sport-specific tasks (40% of studies) and cognitive performance (33% of studies). This suggests that tDCS is a promising tool to improve sportive performance but that it is more effective in no task specific aspects of athletic performance. More importantly, this is the first systematic review to analyze the effects of tDCS on emotional states and cognitive performance in high-level athletes. Emotional state and cognitive performance are crucial for athletic performance under intense conditions. Our analysis showed that tDCS can modulate emotional states, such as improving vigor and decreasing tension, fatigue, and anxiety, as well as cognitive performance, such as improving reaction time and accuracy in decision-making. However, research on the effects of tDCS on emotional states and cognitive performance is relatively sparse. Both of these are key focus points for future research.

tDCS applied to specific brain regions can induce particular neuromodulatory processes (Machado et al., 2021). Through complex networks and systems, several brain areas regulate or promote athletic performance, including the M1, PMC, CB, DLPFC and TC. M1 plays a crucial role in athletic performance, however results with tDCS applied over M1 were inconsistent (Edwards et al., 2017; Machado et al., 2019; Shyamali Kaushalya et al., 2021). Machado et al. (2019) reported that a-tDCS over M1 improved exercise performance in cycling only in healthy adults. Among the seven studies applying tDCS over M1, four showed improvements in muscle strength, sport-specific performance, and emotional states. Regarding muscle strength performance, two studies showed that a-tDCS (2 mA, 20 min) over M1 (C3 or C4) increased the MVIC in handball and football athletes (Hazime et al., 2017; Vargas et al., 2018). tDCS over M1 can enhance corticospinal excitability to alter motor unit firing and muscle recruitment strategies (Krishnan et al., 2014; Dutta et al., 2015; Polania et al., 2018). Regarding sport-specific performance, Park et al. (2022) showed improved volleyball spike speed and consistency using a-tDCS (2 mA, 20 min) over M1. Spike performance is closely related to coordination patterns and muscle synergies of the entire body (Wagner et al., 2014). Veldema et al. (2021) reported that 1-mA a-tDCS over M1 for 20 min improved basketball shooting performance in healthy sports students. This implies that tDCS may have positive effects on specific motor coordination in high-level athletes. In addition, tDCS over M1 may potentially increase explosive force (Lu et al., 2021), as shown for volleyball spike speed. Regarding emotional states, Valenzuela et al. (2019) used 2-mA a-tDCS for 20 min over the M1 and improved vigor in triathletes. A previous study found that tDCS over M1 (C3) improved mood in patients with fibromyalgia, which may be related to elevated serum beta-endorphin levels (Khedr et al., 2017). tDCS has been observed to alter activation and functional connectivity of the region under the anode. Additionally, the conventional method using two large sponge pad electrodes was found to affect the functional connectivity of other cortical and deep brain regions, particularly in the DLPFC (van den Heuvel and Sporns, 2013; To et al., 2018; Piretti et al., 2022). The activation of the DLPFC has been linked to improvements in mood states (Ligeza et al., 2021). Hence, tDCS applied over the M1 likely alter activation and functional connectivity of the DLPFC, potentially causing the observed augmentations in mood after tDCS.

Some studies however report no difference in neuromuscular or sport-specific performance between M1-tDCS and sham stimulation (Mesquita et al., 2019b; Valenzuela et al., 2019; Machado et al., 2021; Liang et al., 2022; Park et al., 2022), suggesting that tDCS over the M1 may not affect sport-specific movement patterns in high-level athletes in each case, and that facilitation of cortical excitability does not always induce improvements in athletic performance in high-level athletes. The brains of high-level athletes show specific neural responses during exercise compared to the general population and non-elite athletes (Del Percio et al., 2010; Nakata et al., 2010; Wright et al., 2010; Casanova et al., 2013; Callan and Naito, 2014; Ermutlu et al., 2015; Monda et al., 2017). Long-term training may develop specialized neural networks that implement the automatic execution of movements and reduce neural activity in high-level athletes (Xu et al., 2016; Guo et al., 2017). These findings are in line with the “neural efficiency” hypothesis, which suggests more efficient cortical functions in more skilled individuals, requiring less cortical activation (Del Percio et al., 2008; Babiloni et al., 2010; Dunst et al., 2014). On the other hand, high-level athletes showed higher baseline levels of neural excitability and brain-derived neurotrophic factor concentration compared to healthy non-athletes (Al-Khelaifi et al., 2018; Donati et al., 2021). Although the exact mechanisms underlying these effects have not been clarified in detail, some studies have shown that tDCS can affect neural excitability and neural plasticity through reducing gamma-aminobutyric acid inhibition and increasing brain-derived neurotrophic factor levels (Liebetanz et al., 2002; Nitsche et al., 2003a; Frazer et al., 2016). Thus, tDCS may not affect on neural excitability and plasticity in high-level athletes. Mesquita et al. (2019b) reported that 1.5-mA a-tDCS over the bilateral M1 for 15 min did not enhance kick frequency in 19 black-belt taekwondo athletes and reduced the total number of kicks compared to a control group. This implied that bilateral M1 stimulation worse performance than unilateral stimulation. This discrepancy may be explained by current flow direction. A recent study reported that corticospinal excitability was decreased after a-tDCS over bilateral M1 in healthy adults (Behrangrad et al., 2022). Studies investigating the effects of tDCS on neural excitability in athletes are scarce. Future research should focus on neural mechanistic studies to elucidate the impact of various protocols and target brain areas of tDCS on high-level athletes.

The PMC forms an important node in frontoparietal networks and contributes to specification of movement parameters (Kantak et al., 2012). One study found that 2-mA a-tDCS over the PMC for 20 min improved MVIC of all upper body muscles and gymnastic performance in gymnastic athletes (Anoushiravani et al., 2023). A recent study showed that a-tDCS over PMC improved functional connectivity within PMC and between PMC and M1, and enhanced motor performance after stroke (Unger et al., 2023). This implies that PMC activation via tDCS could enhance motor network activity and premotor-motor functional connectivity, and result in performance improvements. Further, the CB is an important node in motor control and coordination of complex movements (Jacobs and Horak, 2007). One study found that 2-mA a-tDCS over the CB for 20 min improved MVIC of the right deltoid and trapezius and left biceps and pectoralis muscles and straddle lift to handstand test in gymnastic athletes (Anoushiravani et al., 2023). Kenville et al. (2020) showed that 2-mA tDCS over CB for 20 min improved MVIC during isometric barbell squats in healthy individuals. Studies showed enhanced connectivity in fronto-parietal-cerebellar networks and motor cortex-cerebellar networks following motor adaptation in healthy subjects (Sami and Miall, 2013; Vahdat et al., 2014). tDCS over CB has potential benefits for motor tasks and improves locomotor performance, and tDCS can increase postural control by affecting connections between the CB and M1 and influencing the function of the vermis (Celnik, 2015). There are inhibitory interactions between the CB and M1, which may have limited the effects of tDCS over CB. According to the latest finding, a-tDCS over right CB increased cerebellar brain inhibition and reduced M1 excitability (Galea et al., 2009). Behrangrad et al. found that bilateral a-tDCS over the M1 + CB and bilateral a-tDCS over the CB significantly increased corticospinal excitability (Behrangrad et al., 2022). It seems that the stimulation protocol affects the effects of tDCS over CB on neural excitability and muscle strength enhancement and this should be investigated in future studies.

Five studies included in this review investigated the effects of tDCS applied over the DLPFC on endurance, emotional states, and cognitive performance. Regarding endurance, two studies reported that 1.5-mA HD-tDCS over the bilateral DLPFC (F3 andF4) for 20 min improved total time required for in 2-km TT, PPO and total time required for 15 km distance in cyclists compared with sham stimulation (Pollastri et al., 2021; Gallo et al., 2022). It was reported that 1.5-mA HD-tDCS over the bilateral DLPFC (F3 and F4) for 20 min delayed the increase in ratings of perceived exertion (RPE) during TT and lead to higher power output at the same RPE compared with sham stimulation (Pollastri et al., 2021). The main function of the DLPFC is executive control of behavior (Miller and Cohen, 2001), a-tDCS over DLPFC demonstrated an increase in resting functional connectivity within networks including the DLPFC (Keeser et al., 2011) and increased coupling between the left and right DLPFC and the left sensorimotor cortex (Stagg et al., 2013). A recent study showed that 2-mA a-tDCS over the left DLPFC for 30 min delayed TTE, reduced the heart rate and RPE, and improved Stroop task performance in healthy individuals (Angius et al., 2019). tDCS over the left DLPFC enhances endurance performance, improves inhibitory control, and reduces the perception of effort by enhancing neural activity (Maeoka et al., 2012). Regarding emotional states, Moreira et al. (2021) reported that 2-mA a-tDCS over the left DLPFC for 20 min improved wellbeing scores in soccer players in real competition. Mehrsafar et al. (2020) found that a-tDCS enhanced vigor and reduced tension and pre-competition anxiety in archers. The activation of the prefrontal cortex has been associated with improvements in mood states (Ligeza et al., 2021; Park et al., 2021). It reported that tDCS over left DLPFC promote the descending pain inhibitory system by enhancing neural activity (Maeoka et al., 2012). However, two studies found that tDCS over DLPFC did not improve Stroop task performance in basketball players and cyclists (Gallo et al., 2022; Moreira et al., 2022). The Stroop paradigm is a valid indicator of inhibitory function. Inhibitory control is associated with the exclusion of irrelevant information or behavioral distractions and avoiding errors (Engle, 2016), affecting anticipation and decision-making during competition (Wood et al., 2016). Athletes demonstrate significantly higher cognitive performance than the general population (Nakamoto and Mori, 2008; Post et al., 2009; Irwin et al., 2011; Smith et al., 2016; Andrade et al., 2020). The “neural efficiency” hypothesis proposes that high-level athletes have lower brain activation during simple tasks and only show higher brain activation when performing difficult tasks (Del Percio et al., 2010). Simple cognitive tasks (e.g., Stroop task) may have a “ceiling effect” that does not effectively reflect the effects of tDCS. Besides, laboratory tests lack high levels of ecological validity, which may complicate detection of the effects of tDCS in high-level athletes (Machado et al., 2021). Efficacy of stimulation may also depend on number of sessions. A previous study reported that a-tDCS (2 mA, 20 min) over the left DLPFC over ten consecutive days improved attention and memory in professional athletes (Borducchi et al., 2016). This implies that a single tDCS session may be insufficient to enhance cognitive performance in high-level athletes. Further research should explore the effects of tDCS on cognitive performance in high-level athletes using multi-session and sport-specific cognitive tasks.

Another brain region that plays an essential role in athletic performance regulation is the TC. Two studies included in this review investigated the effects of tDCS applied over the TC on endurance and cognitive function. Regarding endurance, one study found that 2-mA a-tDCS over the left TC for 20 min increased PPO in the MIT in cyclists. The TC is an important part of the central autonomic network, which is involved in higher-order control of autonomic cardiovascular functions (Dono et al., 2020). tDCS over the TC can furthermore increase neural excitability of the insular cortex (Montenegro et al., 2011). The insular cortex is a major contributor to awareness of the body and subjective feelings related to athletes’ perceptions of physical exertion during exercise and is responsible for cardiac autonomic control (Okano et al., 2015; Penna et al., 2021). It has been speculated that a-tDCS over the TC may increase parasympathetic modulation or decrease sympathetic modulation of the autonomic nervous system thereby improving endurance performance (Okano et al., 2015). Regarding cognitive function, one study reported that 2-mA a-tDCS over the middle TC for 20 min improved visuomotor and basketball decision-making skills in basketball players under mental fatigue (Fortes et al., 2022). Previous studies have also demonstrated an association of the TC with perception (e.g., visual search) in exercise (Brascamp et al., 2010; Qiu et al., 2019; Jedidi et al., 2021). Makris and Urgesi. (2015) showed that repetitive transcranial magnetic stimulation over the superior temporal sulcus decreased action anticipation in soccer players. The TC is a node in the action–observation network (Avenanti et al., 2013). tDCS over the TC may activate action-observation network and improve perceptual-cognitive skills in high-level athletes.

The presently reviewed studies contained 14 different electrode placement protocols with eight different reference electrode positions. Holgado et al. (2019) reported the effects of tDCS on exercise performance were not significantly moderated by type of outcome, electrode placement, muscles involved, number of sessions, or intensity and duration of the stimulation. Differences in electrode placement and stimulation protocols also contributed to mixed results. Anoushiravani et al. reported that tDCS over PMC have better effects in improving strength and sport-specific performance of gymnastic athletes in comparison with tDCS over CB (Anoushiravani et al., 2023). Some studies showed that tDCS over DLPFC and M1 yielded inconsistent results in the same swimming test (Valenzuela et al., 2019; Salehi et al., 2022). Athletic performance is influenced by a variety of abilities modulated by different brain regions, and few studies have examined the effects of stimulating different brain regions on identical athletic performance. Kamali et al. (2019) use a two channel a-tDCS, one anode electrode over M1 (Cz, C1 and C2) and one over TC (T3) for 13 min, and found improvements in one-repetition maximum loads in experienced bodybuilders. A recent study reported that corticospinal excitability was significantly higher following tDCS over M1 and DLPFC than M1 (Vaseghi et al., 2015). This implied that dual-site stimulation may have better effects. The effects of different stimulation protocols and dual-site stimulation on high-level athletes should be investigated in future studies.

## 5 Conclusion

A single session of 1.5–2.4-mA tDCS with a duration of 15–30 min improved strength, endurance, sport-specific performance, emotional states, and cognitive performance in national- or international-level athletes in 12 out of the 18 studies. Of these, six refer to the application of tDCS on the motor system (M1, PMC, CB), five on DLPFC and two on TC. Of all the variables examined in our review, the most sensitive to tDCS are strength (improvement in 67% of studies), endurance (improvement in 75% of studies), and emotional states (improvement in 75% of studies). It is interesting to note that the positive effects of tDCS on performance are significantly reduced in the case of sport-specific tasks (40% of studies) and cognitive performance (33% of studies). This suggests that tDCS is a promising tool to improve sportive performance but that it is more effective in no task specific aspects of athletic performance.

In conclusion, our work suggests that a single tDCS session can apply to competition and pre-competition training to improve athletic performance including both physical and psychological aspects of national- or international-level athletes. Given the relevance of tDCS as a tool to improve athletic performance, further studies focusing on various parameters (type of sports, brain regions, stimulation protocol, athlete level and test tasks) and neural mechanistic studies are needed to improve efficacy of tDCS interventions.

## Data Availability

The original contributions presented in the study are included in the article/Supplementary material, further inquiries can be directed to the corresponding author.
